# GRK2 inhibitors, paroxetine and CCG258747, attenuate IgE-mediated anaphylaxis but activate mast cells *via* MRGPRX2 and MRGPRB2

**DOI:** 10.3389/fimmu.2022.1032497

**Published:** 2022-10-06

**Authors:** Monica Thapaliya, Aetas Amponnawarat, John J. G. Tesmer, Hydar Ali

**Affiliations:** ^1^ Department of Basic and Translational Sciences, University of Pennsylvania, School of Dental Medicine, Philadelphia, PA, United States; ^2^ Department of Family and Community Dentistry, Faculty of Dentistry, Chiang Mai University, Chiang Mai, Thailand; ^3^ Departments of Biological Sciences and of Medicinal Chemistry and Molecular Pharmacology, Purdue University, West Lafayette, IN, United States

**Keywords:** GRK2, paroxetine, mast cells, MRGPRX2, FcϵRI, anaphylaxis, MRGPRB2

## Abstract

G protein-coupled receptor (GPCR) kinase 2 (GRK2), which phosphorylates agonist-occupied GPCRs to promote their desensitization, has been investigated as an attractive therapeutic target for cardiovascular and metabolic diseases. Several GRK2-targeted inhibition strategies have been reported including the use of direct pharmacological inhibitors such as paroxetine (a widely prescribed antidepressant) and its analogs such as compound CCG258747. Cross-linking of high affinity IgE receptor (FcϵRI) on mast cells (MCs) and the resulting degranulation causes anaphylaxis and allergic asthma. Using gene silencing strategy, we recently showed that GRK2 contributes to FcεRI signaling and MC degranulation. The purpose of this study was to determine if the GRK2 inhibitors paroxetine and CCG258747 modulate FcεRI-mediated MC responses *in vitro* and *in vivo*. Utilizing rat basophilic leukemia (RBL-2H3) cells and primary mouse lung MCs (LMCs), we found that paroxetine and CCG258747 inhibit FcϵRI-mediated calcium mobilization and degranulation. Furthermore, intravenous administration of paroxetine and CCG258747 in mice resulted in substantial reduction of IgE-mediated passive cutaneous anaphylaxis. Unlike LMCs, human cutaneous MCs abundantly express a novel GPCR known as MRGPRX2 (mouse; MRGPRB2). We found that in contrast to their inhibitory effects on FcεRI-mediated MC responses, both paroxetine and CCG258747 induce calcium mobilization and degranulation in RBL-2H3 cells stably expressing MRGPRX2 but not in untransfected cells. Furthermore, paroxetine and CCG258747 induced degranulation in peritoneal MCs from Wild-type (WT) mice *in vitro* and caused increased cutaneous vascular permeability *in vivo*, but these responses were substantially reduced in *Mrgprb2^−/−^* mice. Additionally, upon intradermal injection, paroxetine also induced neutrophil recruitment in WT but not *Mrgprb2^−/−^* mice. These findings suggest that in addition to their potential therapeutic utility against cardiovascular and metabolic disorders, paroxetine-based GRK2-inhibitors may serve to modulate IgE-mediated anaphylaxis and to enhance cutaneous host defense by harnessing MC’s immunomodulatory property through the activation of MRGPRX2/MRGPRB2.

## Introduction

G protein-coupled receptors (GPCRs) are extensively studied pharmacological targets which account for ~34% of all drugs approved by the US Food and Drug Administration (FDA) (1). Functions of GPCRs are regulated by a process known as desensitization, in which agonist-occupied receptors undergo phosphorylation by GPCR kinases (GRKs) to attenuate the effects of sustained signaling. However, dysregulated GRK2 levels and enhanced desensitization are the main drivers of pathology involved in cardiovascular, renal, metabolic, cancer, and neurodegenerative diseases (2, 3). Extensive efforts have been made to target GRK2 *via* different inhibition strategies that include genetic inhibition, RNA-based aptameric molecules, peptides, and the use of small molecular inhibitors (3).

Paroxetine (Paxil), a widely prescribed FDA-approved selective serotonin reuptake inhibitor (SSRI)-based antidepressant, has been identified as a direct inhibitor of GRK2. It prevents GPCR desensitization by binding to the active site of GRK2 (4), and has been extensively reported to have benefits in osteoarthritis, cardiac hypertrophy, myocardial infarction, and heart failure (4–9). Thus, efforts have been made to develop GRK2-specific inhibitors based on the paroxetine scaffold (10). Compound CCG258747, which has an added indazole ring, has recently been shown to have high selectivity and potency against GRK2 (11).

In contrast to its role in GPCR desensitization, GRK2 contributes to the activation of signaling *via* receptors that do not couple to G proteins. For example, GRK2 is upregulated in human asthmatic lungs (12), it associates with T cell receptors, and T-cell-specific deletion of GRK2 attenuates airway inflammation and hyperresponsiveness in mouse model of allergic asthma (12, 13). Mast cells (MCs) play a central role in the manifestation of allergic responses such as life-threatening anaphylaxis *via* the aggregation of the high affinity IgE receptor (FcϵRI) by allergen (14). One *in vitro* study showed that GRK2 contributes to FcϵRI signaling and mediator release in MCs (15). However, the possibility that paroxetine and CCG258747 can modulate IgE-mediated anaphylaxis has not been tested.

While MCs are characterized by the presence of cell surface FcεRI, a subtype present in the human skin also expresses a novel GPCR, Mas-related GPCR X2 (MRGPRX2; mouse orthologue MRGPRB2) (16–18). Our lab was the first to show that host defense peptides activate MCs *via* this receptor (19) and recent evidence suggests that MRGPRB2-mediated local MC activation plays a crucial role in clearing bacterial infection *via* neutrophil recruitment (20, 21). Additionally, this receptor also contributes to hypersensitivity reactions in response to multiple clinically used FDA-approved peptidergic drugs (22–24). McNeil et al. (16), showed that the presence of a common tetrahydroisoquinoline (THIQ) motif in these drugs contributes to their ability to activate MRGPRX2 and MRGPRB2. Additionally, Wolf et al. (25), showed that cationic tricyclic compounds with hydrophobic center and a halogen substituent such as paroxetine activates MRGPRX2/MRGPRB2. The main purpose of the present study was to determine if paroxetine and CCG258747 inhibit IgE/FcεRI-mediated MC degranulation and anaphylaxis. We found that both paroxetine and CCG258747 inhibit IgE/FcεRI-mediated MC responses *in vitro* and intravenous administration of these compounds in mice results in significant reduction of IgE-mediated anaphylaxis. However, intradermal injection of the compounds activates cutaneous MCs to enhance host defense by harnessing immunomodulatory property through the activation of MRGPRX2/MRGPRB2.

## Materials and methods

### Reagents

All cell culture reagents, 2,4-dinitrophenyalted Bovine Serum Albumin (DNP-BSA; Cat # A23018) and DNP-specific mouse IgE (SPE-7) were purchased from Invitrogen (Gaithersburg, MD, USA). Recombinant mouse interleukin-3 (IL-3) and stem cell factor (SCF) were purchased from Peprotech (Rocky Hill, NJ). 2-Mercaptoethanol (Cat #M7522), p-nitrophenyl-N-acetyl-β-D-glucosamine (PNAG) and Evans blue dye were obtained from Sigma-Aldrich (St. Louis, MO). Fura-2 acetoxymethyl ester was purchased from Abcam (Cambridge, MA, USA). Compound 48/80 was obtained from AnaSpec (Fremont, CA). Phycoerythrin (PE)- conjugated c-kit and Allophycocyanin (APC)-conjugated FcϵRI antibodies were acquired from eBiosciences (San Diego, CA) Paroxetine hydrochloride hemihydrate (CAS RN: 110429-35-1, Product Number: P1977, solvent PBS) was obtained from TCI chemicals, paroxetine analog CCG258747 (solvent DMSO) was kindly provided by Dr. John J. G. Tesmer, Purdue University.

### Mice

All mice were housed in pathogen-free cages on autoclaved hardwood bedding. C57BL/6 (Wild type; WT) mice were purchased from the Jackson Laboratory (Bar Harbor, ME, USA). MRGPRB2 deficient mice (*Mrgprb2^−/−^*) in the C57BL/6 background were generated by the CRISPR-Cas9 core facility of the University of Pennsylvania. Eight-12 weeks mice were used for entire studies. The experiments were approved by the Institutional Animal Care and Use Committee at The University of Pennsylvania.

### Cells

Rat Basophilic Leukemia (RBL-2H3) were cultured in Eagle’s medium (DMEM) supplemented with 10% FBS, L-glutamine (2 mM), penicillin (100 IU/mL), and streptomycin (100 µg/mL) and maintained in monolayer at 37°C with 5% CO_2_. Same culture conditions were used for RBL-2H3 cells stably expressing MRGPRX2 (RBL-MRGPRX2) in the presence of G-418 (1 mg/mL).

### Peritoneal MCs (PMCs)

PMCs from WT and *Mrgprb2^−/−^* mice were isolated and purified as described (26). Briefly, Hank’s Balanced Salt Solution (HBSS) supplemented with 3% fetal bovine serum (FBS) and 1mM HEPES in a 10 mL volume was used to lavage the cells. Cells were then cultured in Gibco Roswell Park Memorial Institute (RPMI) 1640 media with GlutaMAX and 25mM HEPES supplemented with 10% FBS, 5% Non-Essential Amino Acid (NEAA), penicillin (100 IU/mL) and streptomycin (100 µg/mL), 2-mercaptoethanol (45.6 µM), murine IL-3 (10 ng/mL), and murine SCF (30 ng/mL). Non-adherent cells were removed after 48 h and cultured in fresh medium for 4-8 weeks. After 4 weeks of culture > 90% of cells were PMCs as confirmed by alcian safranin staining as described (27).

### Lung MCs (LMCs)

LMCs were obtained from *ex-vivo* differentiation of fresh lung tissue isolated from WT mice as described (28). Briefly, lungs were excised and chopped into fine pieces with scissors. Chopped lungs were then cultured in complete media same as media for PMCs culture, supplemented with 10 ng/mL murine SCF and 10 ng/mL of murine IL-3. Cells coming out from the tissue gets dispersed into the media and after 7-10 days the tissue disappears leaving fat like droplets behind which was strained, and the cells were further cultured for 4-6 weeks with continuous removal of adherent cells 1-2 times a week. The purity LMCs was > 90% as determined by flow cytometry (BD LSR II flow cytometer, BD Biosciences) using anti-c-kit-PE and anti-FcϵRI-FITC antibodies.

### Degranulation

RBL-2H3 cells (5 × 10^4^), LMCs (4 × 10^4^), *Mrgprb2^−/−^* PMCs (1 × 10^4^) were sensitized with DNP-specific mouse IgE (SPE-7) (1 µg/mL). After 16 h, cells were washed with HEPES-buffered saline containing 0.1% BSA, seeded in 96-well clear flat bottom plates, and incubated with different concentrations of paroxetine or CCG258747 (1-30 µM) at 37°C for 30 min and stimulated with antigen (DNP-BSA, 30 ng/mL) at 37°C for another 30 min. For total β-hexosaminidase release, unstimulated cells were lysed in 50 μL of 0.1% Triton X-100. Aliquots (20 μL) of supernatant or cell lysates were incubated with 20 μL of 1 mM p-nitrophenyl-N-acetyl-β-D-glucosamine for 1 h at 37°C. The reaction was stopped by adding 250 μL of a 0.1 M Na_2_CO_3_/0.1 M NaHCO_3_ buffer and absorbance was measured at 405 nm using Versamax microplate spectrophotometer (San Jose, CA, USA). Degranulation in RBL-2H3 cells stably expressing MRGPRX2, WT and *Mrgprb2^−/−^* PMCs was performed similarly with direct stimulation with paroxetine and CCG258747 (0-30 µM) for 30 min.

### Calcium mobilization

RBL-2H3 (1.5 × 10^6^) were sensitized with DNP-specific mouse IgE (1 µg/mL, 16 h). Cells were washed in HEPES-buffered saline containing 0.1% BSA and loaded with 1 μM Fura-2 acetoxymethyl ester for 30 min at 37°C, followed by de-esterification in the buffer for additional 15 min at room temperature. Cells were then incubated with paroxetine or compound CCG258747 (30 µM) for 30 min at 37°C and stimulated with 30 ng/mL of DNP-BSA at 100 sec. For experiments involving RBL-2H3 and RBL-MRGPRX2 cells where the compounds were tested as agonists, cells were stimulated with paroxetine (30 µM) or compound CCG258747 (10 µM) at 100 sec. Calcium mobilization was determined using a Hitachi F-2700 Fluorescence Spectrophotometer with dual excitation wavelength of 340 and 380 nm, and an emission wavelength of 510 nm.

### Evans blue dye extravasation

For IgE-mediated cutaneous anaphylaxis, mice were sensitized *via* intradermal injection with DNP-specific mouse IgE (SPE-7) (20 ng, 30 µL) into the right hind paw or vehicle (PBS, 30 µL) in the left hind paw. After 24 h, mice were given intravenous injection of paroxetine (100 µg, 100 µL, 5 mg/kg) or vehicle (PBS); CCG258747 (100 µg, 100 µL, 5 mg/kg) or vehicle (1% DMSO in PBS) and after 30 min, mice were challenged with an intravenous injection of 100 µg DNP-BSA in 200 µL PBS containing 1% Evans blue for another 30 min. For experiments with paroxetine and CCG758747 mediated cutaneous anaphylaxis, WT and *Mrgprb2^−/−^* mice were intravenously injected with 1% Evans blue in PBS (200 µL) followed by paroxetine (30 µL of 100 µg/mL) or CCG758747 (20 µL of 100 µg/mL) in right hind paw or with respective vehicles (PBS or 1% DMSO) in left hind paw for 30 min. For IgE-mediated anaphylaxis response in *Mrgprb2^−/−^* mice, passively sensitized mice were intradermally injected with same dose of paroxetine and challenged by antigen (20 µg, 200 µL in 1% Evans blue dye in PBS) or vehicle (200 µL in 1% Evans blue dye in PBS) intravenously for 30 min. Mice were then euthanized; paws were removed, weighed, dried overnight, dissolved in 500 µL formamide and incubated at 55˚C overnight. Supernatant was collected and Evans blue dye extravasation was determined by measuring the absorbance at 650 nm.

### Cutaneous neutrophil recruitment

WT and *Mrgprb2^−/−^* mice were intradermally injected with paroxetine (30 µL of 100 µg/mL, 0.1 mg/kg) in right hind paw and vehicle control in the left hind paw. After 3 h, mice were sacrificed and skin tissue from the injection site was harvested for flow cytometric analyses.

### Flow cytometry

To measure the neutrophil recruitment, single cell suspension was prepared from the skin harvested by digesting the skin using the previously published protocol with slight modifications (29). Briefly, the tissues were chopped and digested using digestion buffer (RPMI 1640 containing 0.8% FBS, DNase I (0.15 mg/mL; Roche), collagenase type IV (3.2 mg/mL; Gibco-Life Technologies), and Dispase II (2.6 U/mL; Sigma-Aldrich) for 1 h at 37°C with constant agitation. Further, cells were strained with a 70-µm strainer and red blood cells were lysed with ammonium-chloride-potassium (ACK) buffer and washed with dPBS supplemented with 2% FBS and 10 mM EDTA. Fc receptors were blocked with anti-CD16/32 IgG (BioLegend), and dead cells were detected using a Zombie Yellow Fixable Viability Kit (BioLegend). Cells were then stained with fluorochrome-tagged antibodies to CD45, CD11b, Ly-6G (BioLegend) for measuring neutrophils. Cells stained with FMO were used as needed. Cells were analyzed by a BD LSR II flow cytometer (San Jose, CA) and FlowJo software version 10.7.2 (Tree Star Inc., Ashland, OR).

### Systemic anaphylaxis

Systemic anaphylaxis was performed as previously described (16). Paroxetine (5 mg/kg, 100 µL) or PBS control was injected intraperitoneal in WT mice. Core body temperature was measured with a rectal thermometer (physitemp) every 10 min for 90 min and mice were euthanized after 90 min, and temperature drop from the baseline was calculated.

### Statistical analysis

To ensure scientific rigor and reproducibility, all *in vivo* experiments were performed with 6-10 mice per experiment. All *in vitro* experiments were at least repeated 3-4 times with different batches of cells ran in triplicates. Statistical significance was determined by two-way ANOVA or ordinary one way-ANOVA with multiple comparison test. Significant differences were set at *p ≤ 0.05, **p ≤ 0.01, ***p ≤ 0.001 and ****p ≤ 0.0001 and analyzed by GraphPad Prism version 9.

## Results

### Paroxetine and CCG258747 inhibit FcϵRI-mediated calcium mobilization and degranulation in RBL-2H3 cells

**Figure 1:**
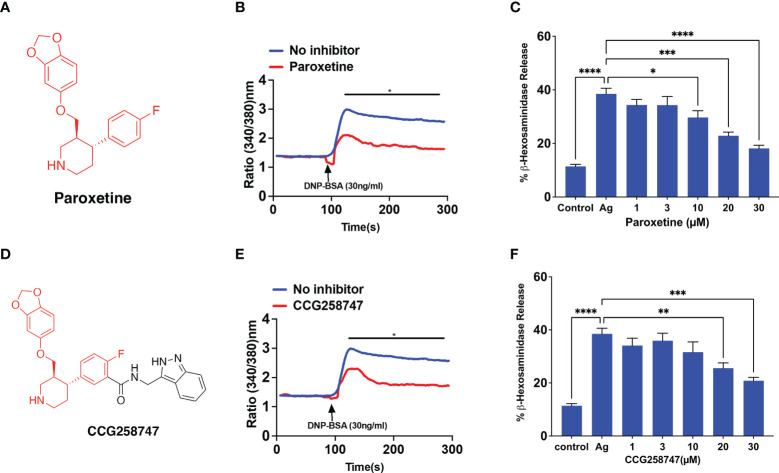
Paroxetine and CCG258747 inhibit FcϵRI-mediated response in RBL-2H3 cells. **(A)** Structure of paroxetine. **(B)** Measurement of Calcium mobilization upon antigen (DNP-BSA; 30 ng/mL) stimulation with/without pre-incubation of IgE-primed RBL-cells with paroxetine (30 µM, 30 min). **(C)** IgE-primed RBL cells were pre-incubated with different doses of paroxetine (0-30 µM) and stimulated with antigen (Ag, 30 ng/mL) and β-hexosaminidase release was measured. **(D)** Structure of compound CCG258747. Measurement of antigen-induced **(E)** calcium mobilization and **(F)** β-hexosaminidase release in IgE-primed RBL-2H3 cells in the absence or presence of CCG258747. Error bars are standard error of mean (SEM) from at least three independent experiments and significant differences were set at *p ≤ 0.05, **p ≤ 0.01, ***p ≤ 0.001 and ****p ≤ 0.0001. Error bars are not displayed for (B) and (E) for clarity. For figure B and E, C and F, data were generated simultaneously from the same experiment with same controls.

RBL-2H3 cells have been extensively used to study the regulation of FcεRI signaling in MCs. Thus, we initially utilized this cell line to determine if GRK2 inhibitor, paroxetine (**Figure 1A**) inhibits FcϵRI-mediated signaling and degranulation. We found that paroxetine at 30 µM inhibits antigen (DNP-BSA)-induced calcium mobilization in cells sensitized with DNP-specific IgE (**Figure 1B**). Next, the inhibitory dose response of paroxetine (0 – 30 µM) on antigen-induced degranulation was measured using a β-hexosaminidase release assay. We found that compared to the control, there was a dose-dependent inhibition of degranulation in paroxetine-treated cells (**Figure 1C**). Likewise, treatment of RBL-2H3 cells with CCG258747 (**Figure 1D**) also resulted in inhibition of IgE-mediated calcium mobilization (**Figure 1E**) and degranulation (**Figure 1F**). These data demonstrate that both paroxetine and CCG258747 inhibit FcϵRI-mediated signaling in RBL-2H3 cells.

### Paroxetine and CCG258747 inhibit IgE-mediated degranulation in primary LMCs *in vitro* and cutaneous anaphylaxis *in vivo*

**Figure 2:**
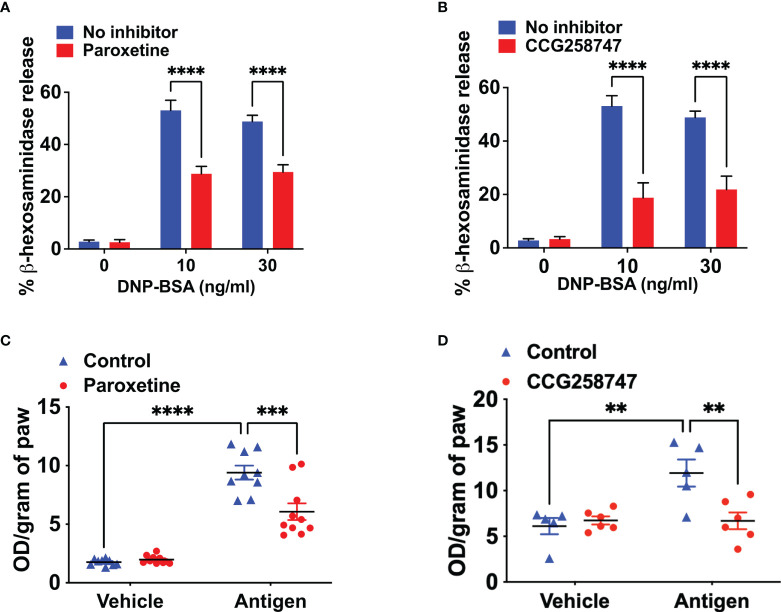
Paroxetine and CCG258747 inhibit IgE-mediated degranulation in primary LMCs *in vitro* and cutaneous anaphylaxis *in vivo*. Measurement of β-hexosaminidase release in IgE-primed LMCs pre-incubated with **(A)** paroxetine (30 µM) and **(B)** CCG258747 (30 µM) and challenged with antigen (30 ng/mL). C57BL/6 mice (n=8-10) were sensitized with IgE (20 ng, 30 µL, right hind paw) and vehicle (PBS, 30 µL, left hind paw) for 24 hrs and were intravenously injected with paroxetine or CCG258747 (5 mg/kg, 100 µL, 30 min) or respective vehicle (100 µL, 30 min) following antigen (20 µg, 200 µL in 1% Evans blue dye in PBS, 30 min) challenge or vehicle (PBS) intravenously. Evans blue dye extravasation was determined. Quantification of vascular permeability for **(C)** paroxetine **(D)** CCG258747. Error bars are standard error of mean (SEM) and significant differences were set at **p ≤ 0.01, ***p ≤ 0.001 and ****p ≤ 0.0001.

Next, we wanted to test if these findings can be translated into a physiologically relevant cell system. For this, we generated murine LMCs and found that paroxetine caused a significant reduction in IgE-mediated degranulation (**Figure 2A**). Treatment of LMCs with CCG258747 also caused a significant reduction of IgE-mediated degranulation (**Figure 2B**). To determine if paroxetine and CCG258747 can inhibit IgE-mediated anaphylaxis *in vivo*, we sensitized mice with intradermal injections with vehicle (PBS, 30 µL, left hind paw) or DNP-specific IgE (20 ng, 30 µL, right hind paw). After 24 h, we injected paroxetine, CCG258747 (5 mg/kg, 100 µL) or vehicle (PBS, 100 µL) intravenously. After 30 min, mice were challenged with intravenous injection of 100 µg DNP-BSA in 200 µL PBS containing 1% Evans blue for 30 min and vascular permeability was assessed by measuring the optical density of dye leakage. Consistent with the *in vitro* data, we found that both paroxetine and CCG258747 treated mice displayed significantly reduced vascular permeability compared to untreated mice (**Figures 2C, D** **)**. These findings confirm that paroxetine and CCG258747 inhibit IgE-mediated MC degranulation *in vitro* and reduce anaphylaxis *in vivo*.

### Paroxetine and CCG258747 induce intracellular calcium mobilization and degranulation *via* MRGPRX2

**Figure 3:**
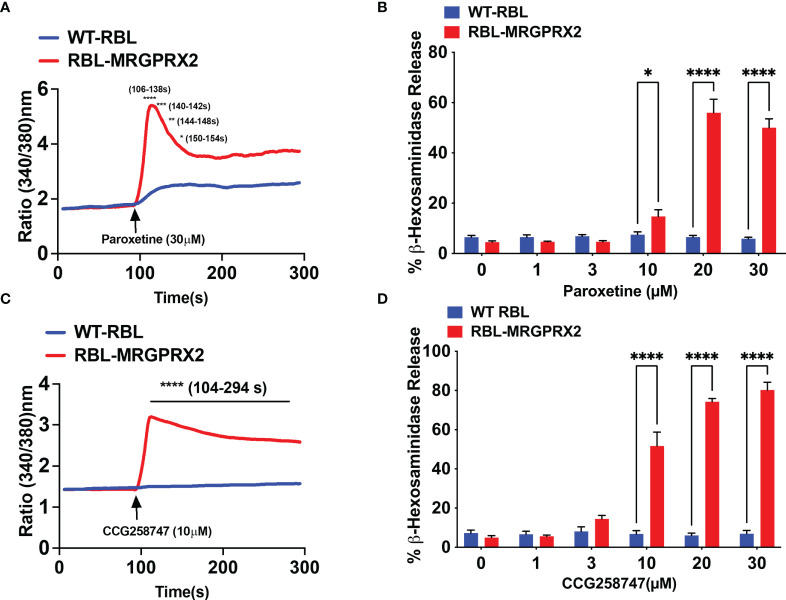
Paroxetine and CCG258747 induces intracellular calcium mobilization and degranulation *via* MRGPRX2. Untransfected RBL-2H3 (WT-RBL) and RBL-stably expressing MRGPRX2 (RBL-MRGPRX2) were exposed to paroxetine and **(A)** calcium mobilization (30 µM) and **(B)** β-hexosaminidase release (0 - 30 µM) were measured. Likewise, CCG258747-induced **(C)** calcium mobilization (10 µM) and **(D)** β-hexosaminidase release (0 - 30 µM) was measured. Error bars are standard error of mean (SEM) from three independent experiments and significant differences were set at *p ≤ 0.05, **p ≤ 0.01, ***p ≤ 0.001 and ****p ≤ 0.0001. Error bars are not displayed for A and C for clarity.

Wolf et al. (25), recently screened a library of pharmacologically active cationic amphiphilic compounds for their ability to activate MRGPRX2 *in vitro* and found that antidepressants desipramine, clomipramine and paroxetine activates MRGPRX2. The authors showed that paroxetine induces calcium mobilization in HEK293 cells transfected with cDNA encoding MRGPRX2 and in a human MC line (LAD2 cells) naturally expressing the receptor with EC_50_ values of 34 µM and 18 µM, respectively. However, the minimum concentration of paroxetine that induces degranulation in LAD2 cells and primary human MCs was 75 µM. The difference between the reported EC_50_ values for calcium mobilization and degranulation is not clear. We found that 30 µM paroxetine caused robust calcium mobilization and maximal degranulation in RBL-2H3 cells stably expressing MRGPRX2 but not in untransfected cells **(** **Figures 3A, B** **)**. The minimum concentration of paroxetine to induce degranulation was 10 µM. We found CCG258747 at 10 µM induced a robust calcium mobilization and substantial degranulation (**Figures 3C, D** **)**. Interestingly, although the dose-response profiles of paroxetine and CCG258747-induced degranulation are similar, the magnitude of the response induced by CCG258747 was greater than paroxetine at all concentrations tested (**Figures 3B, D** **)**. Recently, cryo-electron microscopy structures of agonist stabilized MRGPRX2/G protein complexes have been solved (30, 31). Interestingly, unlike other class A family of GPCRs, MRGPRX2 displays a shallow negatively charged subpocket that facilitates agonist binding and may explain why the receptor is promiscuously activated by positively charged small molecules and peptides. In addition, the receptor contains a second subpocket that facilitates hydrophobic interactions (30). The fact that CCG258747 contains an additional hydrophobic residue (**Figure 1**), could explain why it has a higher efficacy than paroxetine.

### Paroxetine and CCG258747 activate murine MCs *in vitro* and *in vivo via* MRGPRB2

**Figure 4:**
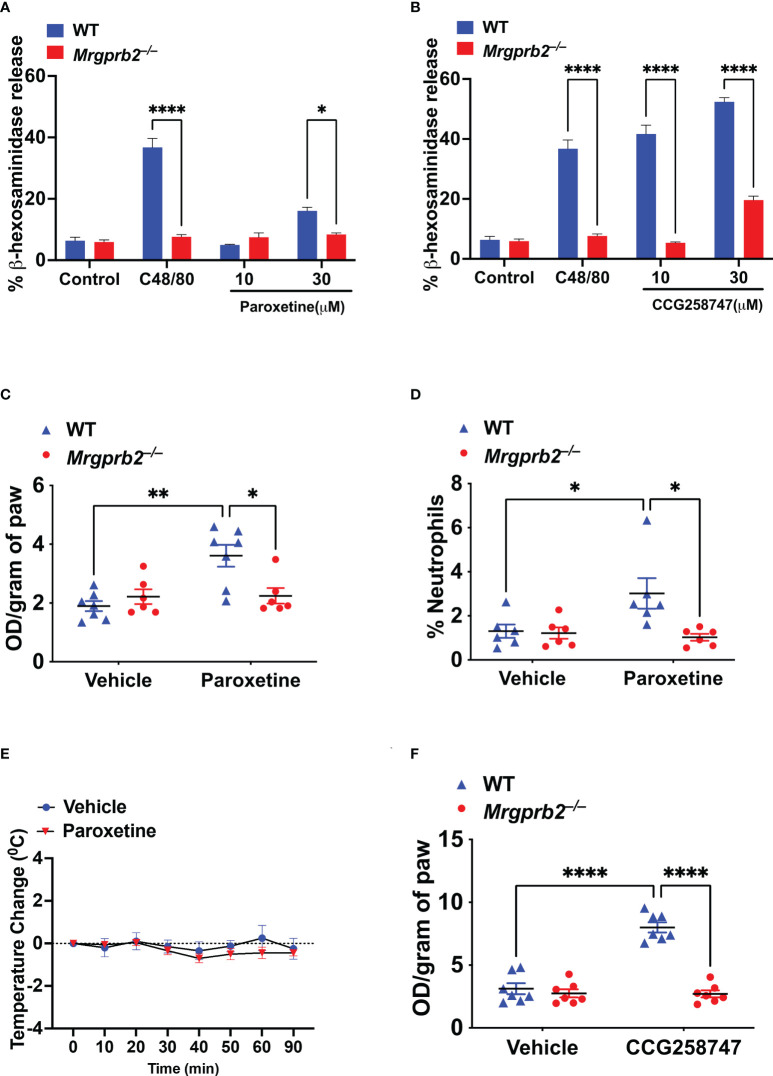
Paroxetine and CCG258747 activate murine MCs *in vitro* and *in vivo via* MRGPRB2. WT and *Mrgprb2^−/−^* PMCs were stimulated with **(A)** C48/80 (10 µg/mL) and paroxetine (10 and 30 µM), or **(B)** C48/80 (10 µg/mL) and CCG258747 (10 and 30 µM), and β-hexosaminidase release was measured. WT and *Mrgprb2^−/−^* (n = 6-7) mice were intradermally injected with paroxetine (0.1 mg/kg, 30 µL, 30 min), **(C)** Evans blue dye extravsation and **(D)** neutrophil recruitment was measured. **(E)** Paroxetine (5 mg/kg, 100 µL) or PBS control was injected intraperitoneal in WT (n=5) mice to assess systemic anaphylaxis *via* rectal temperature drop from baseline. **(F)** Evans blue dye extravasation with intradermal injection with CCG258747 (0.1 mg/kg, 20 µL, 30 min). Error bars are standard error of mean (SEM) and significant differences were set at *p ≤ 0.05, **p ≤ 0.01 and ****p ≤ 0.0001.

Wolf et al., showed that paroxetine induces degranulation in PMCs but the role of MRGPRB2 on this response was not tested. Compound 48/80 (C48/80) is a classic MC degranulating agent that functions *via* MRGPRB2 (16). We first validated *Mrgprb2^−/−^* PMCs by demonstrating that while C48/80 induces robust degranulation in cells cultured from WT mice, this response was abolished in cells cultured from *Mrgprb2^−/−^* mice (**Figures 4A, B** **)**. We then sought to determine if paroxetine and CCG258747 induce degranulation in mouse PMCs and if they did so *via* MRGPRB2. We found that unlike the situation for MRGPRX2, 10 µM paroxetine did not induce degranulation in PMCs but at 30 µM it induced a small but significant response that was not present in *Mrgprb2^−/−^* PMCs (**Figure 4A**). However, unlike paroxetine, 10 µM of CCG258747 induced a robust degranulation response and the compound at 30 µM caused a response that was ~5-fold higher than that induced by the same concentration of paroxetine (**Figure 4B**).

Although Wolf et al. (25), showed that intradermal injection of compounds similar to paroxetine (desipramine, clomipramine) induce scratching behavior in mice *via* MRGPRB2, the effect induced by paroxetine *in vivo* was independent of MRGPRB2. The reason for this difference is not clear. However, it is well documented that MC degranulation *in vivo* is associated with increased local vascular permeability (32). Thus, we tested whether the observed *in vitro* effect of paroxetine and CCG258747 *via* MRGPRB2 can be translated *in vivo* by quantitating injection site vascular permeability. For this, 8-12 weeks of WT and *Mrgprb2^−/−^* mice were injected intradermally with paroxetine (30 µL, 100 µg/mL, right hind paw) or CCG258747 (20 µL, 100 µg/mL, right hind paw) and respective vehicle control (left hind paw) following 1% Evans blue dye intravenously for 30 min, and vascular permeability was assessed by measuring the optical density of dye leakage. Consistent with the *in vitro* data, we found that paroxetine-challenged paw displayed significantly enhanced vascular permeability compared to vehicle control in WT mice, but this effect was significantly reduced in *Mrgprb2^−/−^* mice (**Figure 4C**). Additionally, MRGPRB2 activation contributes to host defense against bacterial infection by releasing MC-mediators and recruiting neutrophils to the sites of infection (21). Evidence shows that paroxetine exerts antimicrobial activity against different bacterial strains (33). Thus, we tested whether paroxetine could recruit neutrophils at the injection site *via* MRGPRB2 activation to harness its immunomodulatory property. Complementing the reduced vascular permeability, paroxetine-treated mice also displayed significantly reduced neutrophil recruitment in *Mrgprb2^−/−^* compared to WT mice (**Figure 4D** **).** MRGPRB2 is expressed in connective tissue-type of murine MCs such as those found in the peritoneum and skin but at low levels in the lung or gut (17, 18). Thus, it was not surprising that paroxetine (5 mg/kg) when injected intraperitoneally did not induce systemic anaphylaxis as measured by temperature drop from baseline **(** **Figure 4E** **).** Similar to paroxetine, CCG258747 challenged *Mrgprb2^−/−^* mice also displayed reduced vascular permeability compared to WT (**Figure 4F** **).** These findings show that both paroxetine and CCG258747 activates MC *via* MRGPRB2 both *in vitro* and *in vivo.*

### Paroxetine inhibits antigen/IgE-mediated degranulation in PMCs *in vitro* and anaphylaxis *in vivo* in the absence of MRGPRB2

**Figure 5:**
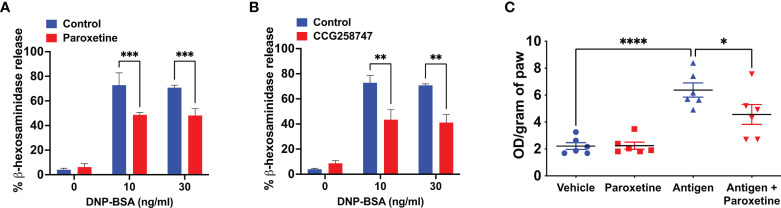
Paroxetine inhibits antigen/IgE-mediated MC degranulation *in vitro* and passive cutaneous anaphylaxis response *in vivo* in *Mrgprb2^−/−^* mice. IgE-primed *Mrgprb2^−/−^* PMCs were preincubated with **(A)** paroxetine (30 µM) and **(B)** CCG258747 (30 µM) and exposed to different concentrations of antigen (DNP-BSA, 0 - 30 ng/mL) and β-hexosaminidase release was measured. Passively sensitized *Mrgprb2^−/−^* mice were intradermally injected with paroxetine (0.1 mg/kg, 30 µL, 30 min, right hind paw) or vehicle (PBS, 30 µL, 30 min, left hind paw) followed by antigen (20 µg, 200 µL in 1% Evans blue dye in PBS, 30 min) or vehicle (200 µL in 1% Evans blue dye in PBS, 30 min) challenge intravenously. **(C)** Evans blue dye extravasation was determined. Error bars are standard error of mean (SEM) and significant differences were set at *p ≤ 0.05, **p ≤ 0.01, ***p ≤ 0.001 and ****p ≤ 0.0001.

Data presented thus far suggest that paroxetine and CCG258747 display dual roles in MCs; in the absence of MRGPRX2 and MRGPRB2 they inhibit IgE-mediated responses but in their presence, they couple to these receptors to induce degranulation. Thus, we wanted to further confirm whether paroxetine and CCG258747 inhibit antigen/IgE-mediated degranulation in *Mrgprb2^−/−^* PMCs. Indeed, we found that both paroxetine and CCG258747 caused significant inhibition of antigen/IgE-mediated degranulation in *Mrgprb2^−/−^* PMCs (**Figures 5A, B** **)**. Additionally, unlike the situation in WT mice, where paroxetine induced increased vascular permeability (**Figures 4C, D** **)**, in *Mrgprb2^−/−^* mice, paroxetine (30 µL of 100 µg/mL) caused significant inhibition of IgE-mediated anaphylaxis as measured by Evans blue extravasation (**Figure 5C**).

## Discussion

Phosphorylation of many GPCRs by GRK2 and the subsequent desensitization provides an important mechanism to prevent detrimental effects of sustained signaling (2, 3). However, in pathologies associated with upregulated GRK2, such as cardiovascular, renal, metabolic, cancer, and neurodegenerative diseases, enhanced receptor desensitization is not always desirable (2, 3). This is the reason why paroxetine-mediated direct inhibition of GRK2 has been reported to be fruitful in mice studies for improving osteoarthritis, cardiac hypertrophy, myocardial infarction, and heart failure (4–8). Furthermore, Tian et al. (9), also showed that paroxetine improves cardiac function in patients which correlates to the reduction of GRK2 expression. Because of this success, efforts have been made to generate more potent and GRK2-specific analogs of paroxetine such as CCG258747 (11). In contrast to its function in GPCR desensitization, GRK2 promotes IgE/FcϵRI mediated MC signaling, which is well-documented in the development and progression of several MC-mediated allergic diseases and hypersensitive reaction (14, 34–37). Utilizing both cell lines and primary MCs, we have shown that paroxetine and CCG258747 inhibit FcϵRI-induced mediator release *in vitro* and anaphylaxis *in vivo.*

In several pre-clinical models and in patients with cardiovascular and metabolic diseases, GRK2 expression and activity is enhanced leading to the disease progression and severity due to enhanced desensitization (5, 38). Although not related to the desensitization function of GRK2, patients with allergic asthma (likely MC-mediated) exhibit GRK2 levels that are enhanced in the lungs compared to healthy controls. Additionally, T-cell-specific GRK2 promotes the pathogenesis of allergic asthma in a mouse model (12). We demonstrated that in mice, paroxetine and CCG258747 treatment reduce IgE-mediated MC degranulation *in vivo*. Thus, the prospect of using paroxetine based GRK2 inhibitors against allergic asthma is an attractive possibility. Furthermore, a randomized control trial also revealed that paroxetine has been successful for therapy against pruritus (39, 40), which is likely mediated *via* MC activation. Thus, in such precedent where paroxetine is clinically approved, paroxetine-based inhibitors may be attractive therapeutics against allergy.

Although CCG258747 is reported to be more potent and specific for GRK2 compared to paroxetine (11), we found that its inhibitory profile for IgE-mediated responses in RBL-2H3 were similar to paroxetine. However, in LMCs, the inhibition was slightly stronger for CCG258747 compared to paroxetine. The reason for this difference is not clear but could reflect differences between a cell line and primary MCs. Nevertheless, the possibility that paroxetine and CCG258747 inhibit IgE-mediated response independent of GRK2 cannot be ruled out. Both compounds are SSRI inhibitors, however, this is an unlikely mechanism because RNA-sequencing analysis revealed that primary LMCs do not express serotonin transporters (unpublished data). There has been a previous postulation that SSRIs suppress IgE-ATP positive feedback *via* purinergic receptors (41), which could be one of the possible mechanisms of action and will be the subject of future studies.

We made an interesting observation that paroxetine and CCG258747 cause MRGPRB2 activation when injected intradermally in mice. However, we did not observe any systemic reaction induced by paroxetine in mice. Furthermore, when injected intravenously, paroxetine did not induce cutaneous anaphylaxis, but instead inhibited FcϵRI-induced response *in vivo.* Paroxetine induces only dermal reaction but not systemic, likely since MRGPRB2 is expressed abundantly in the skin. Nevertheless, the clinical use of paroxetine is oral, and it undergoes metabolism in the gut and liver and its metabolites may have different effects than the parent compound and hence the skin reaction may not be clinically relevant. This could explain why a widely used drug like paroxetine that activates MRGPRX2 does not have many reported allergic reactions (42). Whether paroxetine and CCG258747 induce inflammatory processes is currently unknown. However, very rare cases of dermatological adverse effects of paroxetine such as rash (2%–3%), photosensitivity (2%), and eczema (1%) have been reported (42). Thus, caution may have to be exercised when using these especially in patients who are pre-disposed to co-morbidities where MRGPRX2 is either upregulated (asthma, mastocytosis) or mutated (43–45).

Several reports suggest that SSRI inhibitors such as paroxetine have antimicrobial activity and can be a promising alternative for treating bacterial infection (33, 46, 47). In addition to direct antimicrobial activity, antimicrobial peptides can activate MCs to harness their immunomodulatory properties (26, 48–50). Recent evidence suggests that this immunomodulatory property is mediated *via* activation of MRGPRB2 and it plays a crucial role in host defense and clearing antibacterial infection (19, 20, 22, 26). A surprising observation of our study was that paroxetine evoked an acute inflammatory response by causing increased cutaneous vascular permeability and neutrophil recruitment *via* MRGPRB2. Folleto et al. (33), showed that paroxetine not only has antimicrobial activity but also synergizes with the antimicrobial activity of ciprofloxacin. However, the mechanism of antimicrobial activity of paroxetine is not known. It is possible that this antimicrobial activity of paroxetine is mediated *via* MRGPRX2/MRGPRB2 activation and in addition to its direct antimicrobial property, it can serve to enhance host defense.

In summary, we demonstrated that paroxetine and a related GRK2 inhibitor not only have an attractive clinical prospect in targeting allergic reactions, but also may serve to enhance host defense *via* MRGPRX2/MRGPRB2 activation. These interesting findings of dual regulation have attractive clinical utility, and detailed mechanistic studies in humans are needed to delineate these prospects.

## Data availability statement

The raw data supporting the conclusions of this article will be made available by the authors, without undue reservation.

## Ethics statement

The animal study was reviewed and approved by Institutional Animal Care and Use Committee at The University of Pennsylvania.

## Author contributions

HA contributed to conception, supervision, and funding acquisition of the study. MT contributed to the conception and MT and AA performed the experiments and analyzed the data. MT wrote the first draft of the manuscript. JJGT provided the compound CCG258747 for the experiments. All authors contributed to the article and approved the submitted version.

## Funding

This work was supported by National Institutes of Health grants R01-AI124182, R01-AI143185 and R01-AI149487 to HA, F31 AI154765-01A1 to MT, and R01-HL071818 to JJGT.

## Acknowledgments

We thank the University of Pennsylvania’s CRISPR/Cas9 Targeting Core and Transgenic and Chimeric mouse facility for generating the *Mrgprb2^−/−^* mice for our laboratory.

## Conflict of interest

The authors declare that the research was conducted in the absence of any commercial or financial relationships that could be construed as a potential conflict of interest.

## Publisher’s note

All claims expressed in this article are solely those of the authors and do not necessarily represent those of their affiliated organizations, or those of the publisher, the editors and the reviewers. Any product that may be evaluated in this article, or claim that may be made by its manufacturer, is not guaranteed or endorsed by the publisher.

## Abbreviations

MCs, Mast cells; GPCR, G protein-coupled receptor; GRK2, GPCR kinase 2; MRGPRX2, Mas-related GPCR X2; MRGPRB2, Mas-related GPCR B2; RBL, Rat Basophilic Leukemia; WT, Wild type.
